# Intelligent monitoring and individualized strategies for preventing altitude sickness during altitude training

**DOI:** 10.3389/fphys.2025.1690121

**Published:** 2025-10-21

**Authors:** Longji Li, Tianfei Fan, Zhijie Luo, Peixin Zhu, Lifeng Zhang

**Affiliations:** ^1^ Strength and Conditioning Center, School of Physical Education, Chengdu Sport University, Chengdu, China; ^2^ Department of Pharmacy, West China Hospital, Sichuan University, Chengdu, China

**Keywords:** altitude sickness, altitude training, hypoxic adaptation, intelligent monitoring, personalized intervention

## Abstract

Altitude training is a special training method that uses a hypoxic environment to improve athletic performance. Its scientificity and safety have always attracted much attention. The hypoxic environment at high altitudes causes physiological responses, such as decreased blood oxygen saturation, increased oxidative stress, and changes in vascular permeability. By establishing a multi-level physiological indicator monitoring system, including basic indicator monitoring, special function assessment, and new technology application, the training effect can be effectively evaluated, and the training safety can be guaranteed. In terms of training optimization, the application of individualized programs based on genetic testing and intelligent monitoring systems has significantly improved the scientificity of training. Among them, the step-by-step adaptation method combined with hypoxic pre-training reduce the incidence of acute mountain sickness by 75%, and the individualized program reduce the difference in training effects by 40%. However, altitude training still faces controversial issues such as the ethics of drug intervention and the effect of simulated training. Future research should focus on the gene-environment interaction mechanism, develop new monitoring technologies, and establish a multidisciplinary collaboration system. This review focuses on the physiological mechanisms, monitoring methods, optimization strategies, limitations and future development directions of altitude training in preventing altitude sickness. This provides an important basis for the scientific practice of altitude training. By continuously optimizing the training system, altitude training will become a safe and effective method to improve athletic performance and prevent altitude sickness.

## 1 Introduction

Altitude training is a scientific method that uses hypoxic environments to stimulate physiological adaptation in the body, aiming to enhance athletic performance (Chang et al., 2023). Historically, altitude training can be traced back to the 1968 Mexico City Olympic Games, where athletes first recognized the potential advantages of hypoxic exposure (Suchý and Waic, 2017). In the 1970s, the early concept of “climb high and sleep low” emerged from mountaineering and high-altitude expeditions, reflecting attempts to optimize the balance between hypoxic adaptation and training intensity (Burtscher and Koch, 2016). With the progress of molecular biology and exercise medicine in recent decades, the mechanisms underlying altitude-induced adaptations have been gradually elucidated, providing a stronger scientific rationale for its use. The key mechanism lies in the body’s compensatory physiological responses under high-altitude hypoxia (typically at elevations ≥2000 m), which improve oxygen transport and utilization efficiency, thereby boosting aerobic metabolism (Furian et al., 2022). Athletes engaging in altitude training experienced an approximate 6%–7% increase in maximum oxygen uptake (VO_2_max) and a 5% increase in hemoglobin levels (Chen et al., 2023). The hypoxic environment stimulates the kidneys to secrete erythropoietin (EPO), which promotes bone marrow hematopoiesis, increases red blood cell count and hemoglobin concentration, and improves the blood’s oxygen-carrying capacity (Wiśniewska et al., 2020). Long-term altitude training induces angiogenesis in skeletal muscle, thereby increasing capillary density and facilitating oxygen diffusion and utilization (Lemieux and Birot, 2021). These vascular changes are accompanied by enhanced mitochondrial density and oxidative enzyme activity, which improve aerobic metabolism, delay lactate accumulation, and ultimately contribute to greater endurance performance (Fentaw et al., 2025). In addition, altitude training helps optimize athletic performance. After acclimatizing at high altitude, athletes often return to lower elevations with increased red blood cells and improved oxygen efficiency, leading to better aerobic endurance during competitions (Chen et al., 2023). Intermittent hypoxic training (IHT) involves alternating periods of hypoxic and normoxic exposure. IHT is particularly important for endurance sports such as long-distance running, cycling, and cross-country skiing. It is an effective method to increase VO_2_max and lactate threshold, and also improves high-intensity performance (Huang et al., 2023; Park et al., 2018a; Nakamoto et al., 2016).

Altitude training comes with potential risks, particularly altitude sickness (Gatterer et al., 2024). The risk of developing altitude sickness is influenced by various factors, including individual differences (such as genetics, age, and underlying health conditions), the physiological effects of hypoxia, rate of ascent, and the altitude reached. Altitude sickness mainly includes acute mountain sickness (AMS), high-altitude pulmonary edema (HAPE), and high-altitude cerebral edema (HACE) (Gatterer et al., 2024). AMS is the most common form, affecting approximately 25%–43% of individuals ascending to 2,500–4,300 m and over 60% of those ascending to 6,000 m or higher. It is characterized by symptoms such as headache, nausea, insomnia, and fatigue, which can significantly impair training quality. Symptoms typically appear within 24 h but may develop as early as 6 h or as late as the third day (Zhao et al., 2024). HAPE presents with symptoms like shortness of breath, coughing, and lung crackles. Its incidence is roughly 0.6%–6% at 4,500 m and 2%–15% at 5,500 m. HACE is more severe and may lead to altered consciousness, ataxia, or even coma. It is rarer, with an incidence of about 0.5%–1% at altitudes of 4,000–5,000 m. Both HAPE and HACE can be life-threatening (Berendsen et al., 2022). Under high-altitude hypoxic conditions, reduced blood oxygen saturation, increased oxidative stress, and altered vascular permeability can trigger a series of pathological responses. Therefore, the central challenge of altitude training lies in how to maximize the physiological benefits of hypoxic adaptation while minimizing the risk of altitude sickness.

In order to effectively prevent mountain sickness, scientific adaptive strategies should be adopted for altitude training. Staged Ascent is a common method that gradually improves the body’s tolerance to low oxygen environments by ascending the altitude in stages and following the principle of “climb high and sleep low” (d Almeida, 2023; Burtscher and Tannheimer, 2024). For example, at an altitude of more than 2,500 m, it takes 1–2 days to adapt for every 600 m of elevation. Hypoxic preconditioning is also an effective means (Burtscher M. et al., 2022). Athletes can perform intermittent hypoxic exposure in a simulated hypoxic environment to activate the physiological adaptation mechanism in advance (Behrend et al., 2022). In addition, nutrition and drug intervention play an important role in preventing mountain sickness. Maintaining adequate water and electrolyte balance helps avoid dehydration, while a high-carbohydrate diet can provide rapid energy support (Cao et al., 2025). In terms of drugs, acetazolamide (Diamox) and other drugs relieve the symptoms of mountain sickness by regulating acid-base balance, but they must be used reasonably under the guidance of a doctor (Isakovich et al., 2024). The arrangement of plateau training needs to be optimized according to individual differences. Personalized training plans should be combined with the athlete’s VO_2_max and blood oxygen response to customize the training intensity and duration (Wackerhage and Schoenfeld, 2021). Real-time monitoring is the key to preventing mountain sickness. Through indicators such as blood oxygen saturation (SpO_2_), heart rate variability (HRV), and acute mountain sickness score, abnormalities can be detected in time and the training plan can be adjusted (Koehle et al., 2010). Emergency treatment measures are equally important. Once severe mountain sickness symptoms such as HAPE or HACE appear, it is necessary to immediately descend to a lower altitude area and use a portable hyperbaric oxygen chamber (Gamow Bag) for temporary treatment when necessary (LAWRENCE, 2022).

Although significant progress has been made in the scientific strategies of altitude training, some controversies still exist. For example, there are ethical issues regarding drug interventions, such as the potential health risks of EPO abuse. In addition, the comparison of the effects of altitude training and simulated hypoxia training still requires more research support. Future research directions include the use of genetic markers to predict susceptibility to altitude sickness and the development of smart wearable devices for real-time physiological monitoring. This article focuses on summarizing the scientific strategies for preventing altitude sickness during altitude training, including the pathophysiological mechanisms of altitude sickness, the stepwise adaptation method, hypoxic pre-adaptation technology, nutritional and drug intervention measures, and the principles for the formulation of personalized training programs, providing athletes and coaches with systematic guidance on altitude training safety.

## 2 Pathophysiology of altitude sickness

The impact of high-altitude environments on human physiological function is a complex cascade involving interactions among multiple systems. As altitude increases, the atmospheric oxygen pressure decreases exponentially, leading to hypoxia. This hypoxic condition initiates compensatory and decompensatory responses. A thorough understanding of these mechanisms is essential for the prevention and treatment of altitude sickness.

### 2.1 Physiological effects of hypoxic environments

The hypoxic environment at high altitudes exerts systemic and multi-level effects on the body. Although the fraction of oxygen in air remains approximately 21% at all altitudes, the atmospheric pressure—and therefore the partial pressure of oxygen—decreases exponentially as altitude rises. At an elevation of 3,000 m, the atmospheric oxygen pressure is only about 70% of that at sea level (Ye et al., 2023). This reduction in oxygen partial pressure limits the ability of blood to become fully oxygenated, leading to a significant decrease in arterial oxygen saturation, which may drop below 85%, and can fall below 75% at altitudes above 4,000 m.

Hypoxic conditions cause physiological compensatory mechanisms. The body senses hypoxia via chemoreceptors in the carotid and aortic bodies, stimulating increased ventilation and initiating a “hypoxic ventilatory response” (Bardsley et al., 2023). However, this compensation may lead to respiratory alkalosis, which paradoxically suppresses the ventilatory response, creating a compensatory contradiction (Burtscher J. et al., 2022). Additionally, the cardiovascular system compensates by increasing cardiac output—up to 1.5 to 2 times the sea-level baseline—to maintain tissue oxygenation (Rosales et al., 2022). Nevertheless, these acute compensatory mechanisms are often insufficient to fully counteract hypoxia. At the cellular and molecular level, hypoxia induces excessive production of reactive oxygen species (ROS). When their accumulation exceeds the clearance capacity of antioxidant enzymes such as superoxide dismutase (SOD) and glutathione peroxidase (GPx), oxidative stress ensues (Gaur et al., 2021). This stress condition impairs cellular membrane integrity, disrupts mitochondrial function, and activates inflammatory responses. Studies have shown that in patients with acute mountain sickness, oxidative stress markers such as malondialdehyde (MDA) and ROS are significantly elevated, while antioxidant markers such as SOD and GPx are markedly reduced (Debevec et al., 2020; Irarrázaval et al., 2017; Gong et al., 2018; Sharma et al., 2021). Moreover, hypoxia upregulates the expression of hypoxia-inducible factors (HIFs), thereby promoting the secretion of vascular endothelial growth factor (VEGF) (Jin et al., 2019). Although VEGF facilitates angiogenesis, it also increases vascular permeability, creating conditions conducive to tissue edema. These changes in vascular permeability are particularly evident in the lungs and brain, forming the pathological basis of high-altitude pulmonary edema and cerebral edema.

### 2.2 Classification and symptoms of altitude sickness

Based on clinical manifestations and pathological characteristics, altitude sickness is mainly divided into three categories, including acute mountain sickness (AMS), high-altitude pulmonary edema (HAPE), and high-altitude cerebral edema (HACE), with different severity and prognosis (Savioli et al., 2022). AMS is the most common and mildest type, and the incidence rate increases significantly with increasing altitude. The incidence rate is about 25% at an altitude of 2000–3,000 m, up to 40%–50% at 3,000–4,000 m, and up to more than 60% at more than 4,000 m (Berger et al., 2023). Its typical symptoms include persistent headache (incidence rate of more than 90%), nausea and vomiting (40%–50%), loss of appetite and insomnia. These symptoms usually appear 6–12 h after reaching high altitude and reach a peak in 24–48 h (Berger et al., 2023). It is worth noting that headache is a necessary condition for the diagnosis of AMS. AMS cannot be diagnosed without headache. HAPE is a more serious type, with an incidence rate of about 0.5%–2%, but it can reach 10% in people who quickly ascend to more than 4,500 m (Xu et al., 2021). Its pathological feature is non-cardiogenic pulmonary edema caused by increased pulmonary vascular permeability (Hanaoka et al., 2024). Clinical manifestations include progressively worsening dyspnea (initially only occurs during activity, and later also exists at rest), dry cough (pink foamy sputum may be coughed up in the later stage) and cyanosis. Moist rales can be heard by auscultation, and chest X-ray shows patchy infiltration shadows. If not treated in time, it can rapidly deteriorate within 12–24 h and even lead to death. HACE is the most critical type, with an incidence of about 0.5%–1%, but a mortality rate of up to 40% (Zelmanovich et al., 2022). It is a severe progressive form of AMS, and its pathological features are vasogenic edema caused by increased cerebral blood flow and damage to the blood-brain barrier (Zelmanovich et al., 2022; Li et al., 2025). Clinical manifestations include impaired consciousness (from drowsiness to coma), ataxia (positive straight walking test) and psychiatric symptoms (Li et al., 2023). It is worth noting that HACE patients may also have HAPE, which has a worse prognosis.

### 2.3 Risk factors

The occurrence of mountain sickness is affected by many factors. Understanding these risk factors helps to take targeted preventive measures. In terms of individual differences, genetic factors play an important role. Recent studies have found that polymorphisms of HIF pathway-related genes (such as EPAS1 and EGLN1) are closely related to susceptibility to mountain sickness (Yang et al., 2021). Underlying diseases such as chronic cardiopulmonary disease, anemia and sleep apnea will significantly increase the risk of disease (Geng et al., 2025; Chatterjee and Gupta, 2023). Ascent speed and altitude are the most critical environmental factors. Studies have shown that when the daily ascent speed exceeds 300 m, the incidence of AMS increases significantly. In terms of altitude, 2,500 m is an important threshold (Simancas-Racines et al., 2018). After exceeding this height, the incidence rate increases exponentially with increasing altitude. In addition, factors such as cold (increase oxygen consumption), dehydration (increase hemoconcentration), and strenuous exercise (increase oxygen debt) will also synergistically increase the risk of disease (Vartika et al., 2022). It is worth noting that a history of altitude sickness is the strongest predictor. The risk of recurrence in people with a history of HAPE is 5–10 times that of the general population (Gatterer et al., 2024). Therefore, more stringent preventive measures and monitoring programs must be taken for high-risk groups.

## 3 Adaptive strategies for altitude training

As an important means to improve sports performance and adaptability, the scientificity and systematicity of altitude training are directly related to the training effect and safety. With the in-depth research on sports medicine and hypoxia physiology, the adaptive strategies for altitude training have formed a complete theoretical system and practical programs. These strategies are not only applicable to professional athletes, but also have important guiding significance for the plateau adaptation of the general population. According to existing research and practical experience, the adaptive strategies for altitude training mainly include three aspects: step-by-step adaptation, hypoxia pre-adaptation, and nutrition and drug intervention.

### 3.1 Step-by-step acclimatization method

The step-by-step acclimatization method is a systematic training method developed based on the physiological adaptation patterns of the body to a low-oxygen environment. This method emphasizes the gradual process of altitude adaptation, and achieves safe and effective adaptation through the principle of “climb high and sleep low” (d Almeida, 2023). In specific implementation, differentiated adaptation plans need to be formulated according to different altitude intervals. The currently recognized altitude step training guidelines emphasize the principle of gradual progress. First of all, a gradual ascent should be adopted as much as possible to avoid rapid ascent to areas with an altitude of ≥3,000 m. When the altitude exceeds 3,000 m, the daily ascent height should be controlled within 300–500 m, and an additional day of acclimatization time is required for every 1,000 m of ascent. In addition, before climbing to higher altitudes (such as above 4,000 m), it is recommended to stay in a medium altitude area of 1,500–2,500 m for 4–6 days to ensure that the body is fully adapted to the low-oxygen environment (Richalet JP. et al., 2021). This scientific strategy effectively reduces the risk of acute altitude sickness and improve plateau adaptability.

This phased adaptation training avoids the acute stress response caused by sudden exposure to severe hypoxic environment, and the gradual rise is conducive to the body to gradually establish an effective compensatory mechanism. Research data show that the incidence of AMS can be controlled between 12%–15% for trainees who adopt the standard step-by-step adaptation program, while that of those who are not adapted is as high as 58%–62% (Berger et al., 2020). During the adaptation process, the body will undergo a series of characteristic physiological changes. Blood oxygen saturation drops rapidly to 80%–85% within the initial 24–48 h, and then gradually recovers to more than 90% through ventilation compensation within 3–5 days (Waldner et al., 2025). Hemoglobin concentration shows a biphasic change, with a short-term increase due to blood concentration in the early stage, and then a real increase of 10%–15% within 2 weeks under the action of EPO(18). Mitochondrial function is temporarily inhibited within 1 week, and the density increases by 20%–30% after 4 weeks, and the oxidase activity is significantly improved (Mohanty and Ahmad, 2025). These adaptive changes together constitute the body’s physiological reserve for hypoxic environment.

While the “climb high and sleep low” method is widely used for safe acclimatization, alternative strategies such as “live high, train low” (LHTL) have also been studied to optimize training intensity while maintaining altitude-induced adaptations. This model involves living at moderate to high altitudes (1,250–3,000 m) while performing high-intensity training at lower altitudes (0–1,200 m) to maximize low-oxygen adaptation without compromising training quality. Recent studies support its benefits for endurance athletes. For example, Bonato et al. reported improvements in VO_2_max, time-trial performance, and peak power output with LHTL(51). Therefore, LHTL should be applied with individualized adjustments and consideration of multiple factors.

### 3.2 Hypoxia pre-adaptation training

Hypoxia pre-adaptation training is an important part of the modern altitude training system. Its core is to induce physiological adaptation in advance by simulating the plateau environment (Wang et al., 2024). At present, there are three types of pre-adaptation methods: normobaric hypoxia chamber training, hypoxia sleep system and Intermittent hypoxic training (IHT). Normobaric hypoxia chamber training usually adopts the “3-5-90” scheme, that is, 3–5 times a week, 90 min each time, simulating an altitude of 2,500–3,000 m, combined with intermittent exercise (Chang et al., 2023; Deldicque, 2022); the hypoxia sleep system recommends the “8–2000” mode, which is exposed to a simulated 2000–2,500 m environment for 8 h at night, which can significantly increase EPO secretion (Deng et al., 2025); IHT adopts a “1:1” time ratio, alternating between simulating 3,000 m hypoxia and normoxic environment (Behrend et al., 2022; Park et al., 2018b; Huang et al., 2023). This mode is particularly conducive to promoting angiogenesis.

After 2–4 weeks of systematic pre-adaptation training, trainees achieve multiple physiological improvements. The hypoxic ventilation response slope increased from the basic 0.3–0.5 L/min/%SpO_2_ to 0.5–0.8, an increase of 30%–50% (Xie et al., 2024; Richalet and Gore, 2008; Ross et al., 2023). Muscle capillary density increased by 15%–20% through VEGF-mediated angiogenesis. The antioxidant defense system was significantly enhanced, and the activity of SOD and glutathione peroxidase (GSH-Px) increased by 40%–60% (Xie et al., 2024; Luo et al., 2022). These changes brought about significant functional improvements. VO2max increased by 5%–8% under simulated altitude and 8%–12% in actual plateau environment (Richalet and Gore, 2008). The blood lactate-speed curve shifted to the right, and the exercise intensity at the 4 mmol/L threshold increased by 10%–15% (Xie et al., 2024; Supriya et al., 2022). Subjective fatigue (RPE) decreased by 1–2 levels under the same load. It is worth noting that there are obvious individual differences in the pre-adaptation effect, which is closely related to the HIF-1α gene polymorphism. Therefore, it is recommended to design a personalized program under professional guidance.

### 3.3 Nutrition and drug intervention

Scientific and complete nutritional support and reasonable and necessary drug intervention are important guarantees to ensure the safety and effectiveness of altitude training. In terms of nutritional management, a supplementation system needs to be established. The first is water replenishment (Viscor et al., 2023). It is recommended to use the “weight + environment” formula to calculate the water requirement (35 mL/kg + 500 mL/1000 m), maintain urine volume>1,500 mL/d and urine specific gravity<1.020. Electrolyte supplementation should follow the “sodium-potassium balance” principle, sodium 60–80 mmol/L, potassium 20–30 mmol/L, and pay special attention to magnesium supplementation (400 mg/d) (Bradbury et al., 2020b; Jeukendrup, 2014). Carbohydrates should adopt a “double high” strategy, accounting for more than 70% of total calories (5–7 g/kg/d), and high GI foods should be supplemented before and after training. Antioxidant network construction requires maintenance (Askew, 2002; Koivisto et al., 2019).

In terms of drug intervention, acetazolamide (Diamox) is still the gold standard drug, and its use needs to follow the “three-level prevention” principle (Luks et al., 2024; Wang et al., 2025). Basic prevention (125 mg bid, starting 24 h before ascent) reduces the risk of AMS from 40% to 20%. The therapeutic dose (250 mg bid) can relieve symptoms within 12–24 h. For people at high risk of HAPE, combined with nifedipine sustained-release tablets (30 mg qd) can be considered (Deshwal et al., 2012; Berger et al., 2022; Spooner et al., 2007). Other auxiliary drugs include: dexamethasone (4 mg q6h) for rapid ascent scenarios (Clark and Sheraton, 2025), ginkgo leaf extract (80mg/q12h) to improve microcirculation (Murdoch, 2010), and iron (360 mg ferrous fumarate/qd) to promote hematopoiesis (Garvican-Lewis et al., 2016). It is particularly important to emphasize the standardization of drug intervention, which must be evaluated before medication (renal function, electrolytes, drug allergy history), monitored during implementation (daily symptom score, urine routine), and evaluated for effectiveness (symptom improvement rate, changes in physiological indicators).

A large number of clinical studies have confirmed that this comprehensive intervention program achieves significant results. The incidence of AMS is reduced by 40%–50%, the severity of symptoms is reduced by 30%, the exercise endurance index is improved by 15%–20%, and the physiological adaptation time is shortened by 30%–40% (Wang et al., 2025; Gao et al., 2021; Sridharan and Sivaramakrishnan, 2018). However, it must be noted that any drug intervention must be carried out under the guidance of a professional physician, and special attention should be paid to the contraindications and interactions of various drugs. For example, acetazolamide is contraindicated for those who are allergic to sulfonamides, and dexamethasone is not suitable for diabetic patients.

## 4 Monitoring and evaluation of altitude training

A scientific system for monitoring and evaluating altitude training is essential to ensure both the safety and effectiveness of the training process. By establishing a complete physiological indicator monitoring system, standardized effect evaluation methods and strict safety measures, we can fully grasp the athletes’ adaptation status, adjust training plans in a timely manner, and maximize the benefits of altitude training. Modern altitude training monitoring has evolved into a multidisciplinary and integrated framework involving fields such as sports medicine, hypoxic physiology, and biomechanics. This system not only focuses on immediate physiological responses but also evaluates long-term adaptation outcomes, while establishing a robust safety warning mechanism.

### 4.1 Physiological indicator monitoring system

Altitude training has established a complete set of physiological indicator monitoring system, which includes three levels: basic indicator monitoring, special function evaluation and new technology application (Girard et al., 2013). Basic indicator monitoring is the most important routine monitoring content in daily training. It adopts an all-weather dynamic tracking mode and mainly includes the following key indicators. Blood oxygen saturation monitoring requires the use of a certified medical-grade pulse oximeter (Dünnwald et al., 2021; Lauterbach et al., 2021). Measurements are taken at four time points: in the morning, before training, during training and after training. Before each measurement, you need to rest for 5 min and record the average of three measurements (Tannheimer and Lechner, 2019). Ideally, at an altitude of 2,500 m, the athlete’s resting blood oxygen saturation should be maintained above 88% (Bradbury et al., 2020b). Heart rate variability monitoring collects data through professional heart rate belt equipment, analyses indicators such as SDNN and RMSSD, and evaluates the regulatory function of the autonomic nervous system. Under normal circumstances, athletes who are well adapted to the plateau should maintain their HRV indicators above 80% of the plain value (Altini, 2020; Altini et al., 2020). Fluid balance monitoring includes daily morning weight measurement (fluctuation range controlled within ±1%), urine specific gravity test (maintained between 1.010–1.025), and urine biochemical analysis (focusing on urine protein and urine ketone body indicators) (Armstrong et al., 2025; Wardenaar, 2022). These basic indicators require the establishment of personal files and the drawing of change trend charts to facilitate timely detection of abnormal conditions.

Specialized assessments require the use of professional equipment and testing methods, mainly including VO_2_max test in a hypobaric oxygen chamber and hemoglobin quality test. The VO_2_max test in a hypobaric oxygen chamber precisely controls the oxygen concentration (simulating an altitude gradient of 1,500–3,500 m), adopts a standardized incremental load scheme (increasing 35W power every 2 min), and simultaneously monitors changes in oxygen uptake, ventilation, and blood lactate to draw an aerobic capacity curve in a plateau environment (Wachsmuth et al., 2013; Mujika et al., 2024). The CO rebreathing method is recommended for hemoglobin quality testing. This method accurately calculates the total amount of circulating hemoglobin by measuring the binding capacity of carbon monoxide, avoiding the errors caused by changes in plasma volume in traditional blood tests (Garvican-Lewis et al., 2015; Schmidt et al., 2020). The test frequency is recommended to be once a week. After 4 weeks of plateau training, an increase of 5%–8% in hemoglobin mass indicates good adaptation. In addition, regular muscle biopsies (once before and after training) are required to observe changes in mitochondrial density and oxidase activity, as well as bone density scans (once a month) to evaluate the impact of the plateau environment on the skeletal system (Hadanny et al., 2022).

With the rapid advancement of wearable technology, intelligent portable devices have become increasingly important for real-time monitoring during altitude training. These devices can continuously capture key physiological parameters such as heart rate, blood oxygen saturation, respiratory rate, and physical activity. Their advantages lie in portability, non-invasiveness, and the ability to provide long-term, field-based data collection under real training conditions. Real-time feedback from these devices allows athletes and coaches to adjust training loads promptly, thereby improving both the safety and effectiveness of altitude training. Through the linkage analysis of multi-parameter sensors (blood oxygen, heart rate, skin temperature, acceleration) combined with machine learning algorithms, the risk of acute mountain sickness can be predicted 12–24 h in advance, with an accuracy rate of more than 85% (Wei et al., 2022). For example, commercially available wearable devices such as Garmin Forerunner 955, Polar Vantage V2, Apple Watch Ultra, and medical-grade systems like Zephyr BioHarness and Hexoskin can continuously monitor SpO_2_, HR, and motion parameters in real time. On the algorithm side, recurrent neural networks, particularly long short-term memory (LSTM) models, have shown strong predictive power in time-series physiological data, and can be implemented using open-source frameworks such as TensorFlow (https://www.tensorflow.org/), PyTorch (https://pytorch.org/), or Keras (https://github.com/keras-team/keras/tree/master/examples). Moreover, open databases such as PhysioNet (https://physionet.org/) and publicly available repositories on GitHub provide accessible training and validation resources for these models, while commercial platforms like WHOOP and Firstbeat Analytics already integrate similar physiological monitoring for sports and high-altitude adaptation.

Complementing wearable-based monitoring, advanced laboratory technologies provide in-depth insights into the physiological and molecular mechanisms underlying hypoxic adaptation. Near-infrared spectroscopy (NIRS) technology can non-invasively and in real time monitor changes in the oxygenation status of specific muscle groups (such as the vastus lateralis) during exercise (Perrey et al., 2024). The sampling frequency can reach 10 Hz, which can accurately reflect the oxygen supply and demand balance of local muscles. In addition, portable brain oxygen monitors can observe the oxygenation of brain tissue in real time, providing early warning for the prevention of high-altitude cerebral edema (Si et al., 2021). Wireless electromyography systems synchronously monitor the activation patterns of multiple muscles and evaluate the impact of the plateau environment on neuromuscular control (Ngo et al., 2022). Data from these advanced technologies are integrated and analyzed through cloud platforms to generate personalized adaptation status curves and training recommendation reports, offering precise mechanistic insights that are not achievable with wearable devices alone.

Importantly, in practice, wearable devices (e.g., SpO_2_, heart rate, sleep monitoring) are advantageous for their portability and feasibility in real-world high-altitude training, allowing continuous non-invasive surveillance. By contrast, advanced laboratory techniques such as NIRS, brain oxygen monitors and wireless electromyography systems provide more precise mechanistic insights but require specialized equipment and controlled environments. Therefore, wearable-based monitoring and laboratory-based assessments should be viewed as complementary approaches: the former ensures practical, field-based safety and adaptation tracking, whereas the latter deepens our understanding of underlying physiological mechanisms when conditions permit.

### 4.2 Evaluation criteria for adaptation effect

**FIGURE 1:**
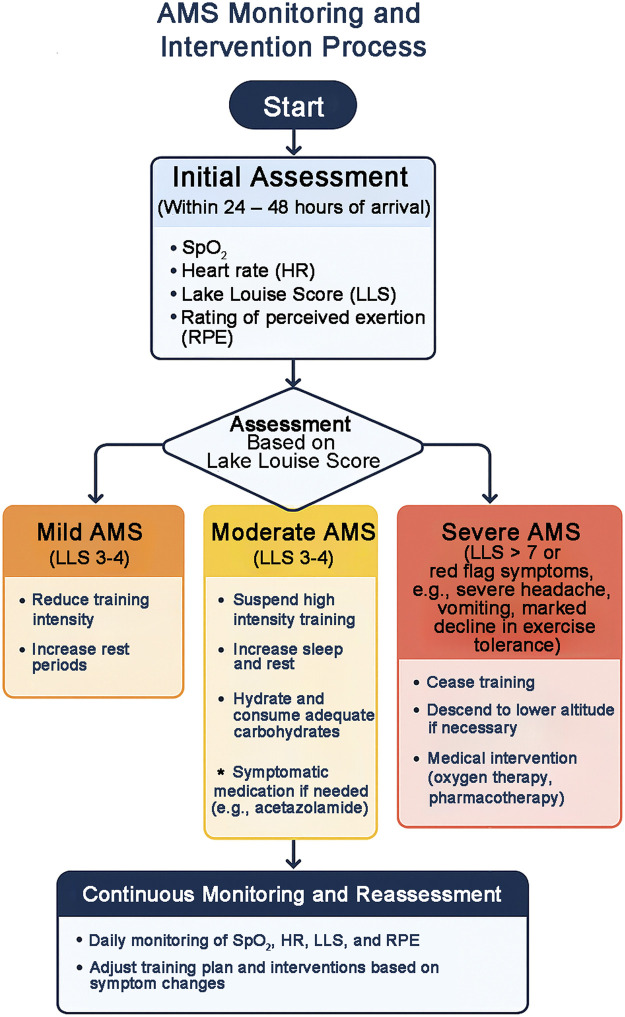
The flowchart of AMS monitoring and intervention during altitude training, based on Lake Louise Score thresholds and physiological indicators (SpO_2_, HR, RPE). Mild AMS (3–4 points) requires reduced training intensity and more rest, moderate AMS (5–6 points) requires suspension of training and medical observation (with possible O_2_ therapy), and severe AMS (≥7 points) requires immediate descent and emergency treatment.

The evaluation of the adaptation effect of altitude training requires the establishment of a systematic and standardized evaluation system, which includes three dimensions: symptom evaluation, physiological adaptation evaluation and sports performance evaluation. The internationally accepted Lake Louise Rating Scale (2018 revised version) is the gold standard for evaluating the symptoms of acute mountain sickness (Roach et al., 2018; Richalet J-P. et al., 2021). The scale includes five symptom dimensions: headache (0–3 points), gastrointestinal symptoms (0–3 points), fatigue (0–3 points), dizziness (0–3 points) and sleep disorders (0–3 points), as well as a functional evaluation dimension: straight line walking test (0–3 points). The scoring criteria are: 3-4 points for mild acute mountain sickness, which requires adjustment of the training plan; 5-6 points for moderate, which requires suspension of training and medical observation; 7 points or more for severe, which requires immediate descent for medical treatment (Figure 1). This evaluation should be completed within 1 h after getting up in the morning, and standardized operations should be performed by professional medical personnel to ensure the comparability of the evaluation results. It is worth noting that the new version of the scale adds nighttime blood oxygen monitoring data as an objective reference indicator, making the evaluation more comprehensive and accurate.

Quantitative evaluation of physiological fitness is the core content of the evaluation of plateau training effects. The EPO response curve is an important indicator for evaluating the adaptation of hematopoietic function (Baranauskas et al., 2022; Tong et al., 2016; Man et al., 2021). The ideal plateau acclimatizer should have an EPO level increase of more than 50% within 24 h after arriving at the plateau, reach a peak value (2–3 times the baseline) in 48–72 h, and gradually fall back to a level slightly higher than the baseline after 7–10 days. This dynamic change process can be drawn through blood tests three times a week to draw a complete response curve. Another key indicator is ventilation sensitivity, which is evaluated by hypoxic ventilatory response (HVR) test, using standardized hypoxic mixed gas (12% oxygen concentration) to measure the slope of minute ventilation (Horiuchi et al., 2022; Bhaumik et al., 2003). The HVR slope of those who are well adapted to the plateau should be between 0.5–0.8 L/min/%SpO_2_. In addition, it is necessary to monitor the adaptation of the antioxidant system, including indicators such as SOD activity, GSH-Px activity and total antioxidant capacity (T-AOC) (Oeung et al., 2023; Constantini et al., 2021; Płoszczyca et al., 2018). These indicators should increase by 30%–50% after 4 weeks of plateau training to indicate good antioxidant adaptation. The pre-evaluation measurements are conducted after an initial acclimatization period of 24–48 h following arrival at altitude, to minimize the influence of acute hypoxic responses and ensure participant safety. This period balances the need for stable baseline measurements with logistical considerations, and assessments are conducted under professional medical supervision.

Sports performance evaluation requires the development of personalized test plans in combination with the characteristics of the project. For endurance athletes, the comparison of plateau/plain results is the most direct evaluation method (Yu et al., 2022). After 4 weeks of training at an altitude of 2,500 m, the difference between the special endurance test results (such as 3000-m run) and the plain should be reduced to less than 3%. The blood lactate-power curve is an objective indicator for evaluating the improvement of aerobic capacity. In a standardized incremental load test (power bike or treadmill), the power or speed corresponding to the 4 mmol/L lactate threshold should increase by 5%–8% after plateau training (Chen et al., 2023; Diebel et al., 2017; Dragos et al., 2022). In addition, recovery ability is also an important evaluation content. By measuring the half-life of blood lactate clearance after standardized exercise load (such as 80% VO_2_max intensity exercise for 10 min), the clearance rate of those who are well adapted to the plateau should increase by 20%–30% (Bonato et al., 2023; Huang et al., 2025). These sports performance indicators need to be correlated with physiological adaptation indicators to establish a prediction model to provide a scientific basis for the formulation of personalized training plans. All evaluation data should be processed using professional statistical methods (such as repeated measures analysis of variance, multivariate linear regression, etc.) to ensure the scientificity and reliability of the conclusions.

### 4.3 Medical safety guarantee

The medical safety guarantee for plateau training requires the establishment of a complete three-level warning system and emergency response mechanism. The graded warning system sets three response levels according to the abnormal degree of physiological indicators (Clark and Sheraton, 2025; Aksel et al., 2019). The first-level warning (yellow) is for the situation where the resting SpO_2_ is between 80%–85% and the Lake Louise score is 3–4 points. The treatment measures include adjusting the training intensity, increasing the rest time and low-flow oxygen therapy. The second-level warning (orange) is for the situation where the SpO_2_ is between 75%–80% and the Lake Louise score is 5–6 points. It is necessary to immediately suspend training, absolutely rest in bed, medium-flow oxygen therapy (2–4L/min) and drug treatment (acetazolamide 250 mg bid). The third-level warning (red) is for situations where SpO_2_ is less than 70% and HAPE or HACE symptoms appear. Emergency evacuation procedures must be initiated, and high-flow oxygen therapy (6–8L/min) and emergency drugs (nifedipine 10 mg sublingual or dexamethasone 8 mg iv) must be given at the same time. This warning system needs to operate 24/7 and is implemented by a dedicated medical monitoring team. All warning events must be recorded in detail and the causes analyzed.

The construction of the medical station at the plateau training base needs to meet strict standards. The site requirements include an independent diagnosis and treatment area (at least 20 square meters), an observation room (equipped with 2–3 beds) and a treatment room, which should be located within a 5-min walk of the training site (Luks et al., 2024). The equipment configuration must include: diagnostic equipment (portable ultrasound, 12-lead electrocardiograph, fully automatic biochemical analyzer), treatment equipment (transport ventilator, defibrillator monitor, infusion pump) and special equipment (portable hyperbaric oxygen chamber, mobile hypothermia therapy device) (DiMM et al., 2024). The drug reserves need to meet the emergency needs of 10 people, including specific drugs for altitude sickness (acetazolamide, dexamethasone, nifedipine), emergency drugs (epinephrine, atropine, amiodarone) and conventional drugs (antibiotics, antipyretic analgesics) (Omidi et al., 2025). The medical team should be composed of a specialist in altitude sickness (at least 1), a sports medicine physician and an emergency nurse. All personnel must hold a certificate of high altitude emergency training and participate in emergency drills every quarter. The medical station needs to establish a 24-h duty system to ensure that timely medical services can be provided at any time (Berger et al., 2020).

The multidisciplinary emergency response process emphasizes the principle of “rapid assessment and graded treatment” (Wolf and Gaddy, 2025; van Veelen et al., 2025). The standardized emergency process includes four key links: on-site assessment (complete vital sign measurement and preliminary diagnosis within 5 min), oxygen therapy initiation (establish an effective oxygen therapy pathway within 10 min), drug intervention (give targeted drug treatment within 15 min) and descent transfer (start the descent procedure within 30 min). For HAPE patients, semi-recumbent position, high-flow oxygen therapy and nifedipine should be adopted; for HACE patients, the head should be kept high and the feet should be low, dexamethasone should be used, and mannitol dehydration treatment should be considered (Savioli et al., 2022). All emergency treatments should follow the principle of “saving lives first, then treating diseases”, and give priority to ensuring the stability of vital signs. The descent transfer requires pre-planning of routes and transportation to ensure that it can be carried out under any weather conditions. During the descent, a clinical assessment should be conducted every 500 m until the symptoms are significantly improved (Omidi et al., 2025). In order to improve emergency response capabilities, it is recommended to conduct a full-element emergency drill every quarter to simulate various possible emergency situations and test the feasibility and effectiveness of the emergency plan. At the same time, a two-way referral mechanism should be established with tertiary hospitals in low-altitude areas to ensure that critically ill patients can receive timely advanced life support.

## 5 Optimization methods for training arrangements

**FIGURE 2:**
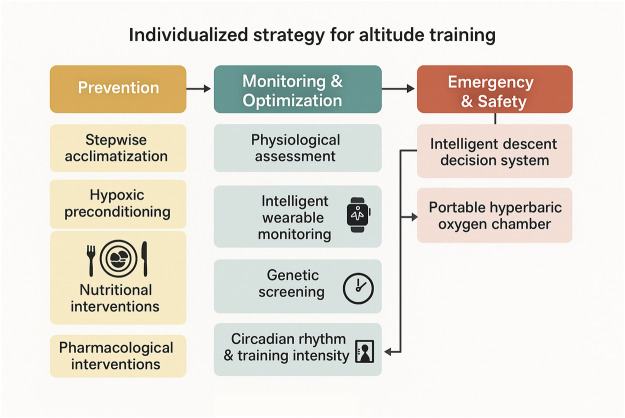
Individualized strategy for altitude training. The multi-tiered framework integrates genetic screening, physiological assessment, stepwise acclimatization, hypoxic preconditioning, nutritional and pharmacological interventions, and intelligent wearable monitoring to optimize training outcomes and minimize altitude sickness risk. Nutritional interventions focus on electrolyte, iron, and protein supplementation, while pharmacological interventions include acetazolamide, dexamethasone, and iron therapy when indicated.

The scientificity and effectiveness of altitude training depend largely on the degree of optimization of the training program. With the in-depth research on sports science and hypoxia physiology, the arrangement of altitude training has developed from the traditional empirical type to the precise and intelligent direction. Modern altitude training optimization methods integrate multidisciplinary knowledge such as molecular biology, sports biomechanics, and environmental physiology. By establishing a standardized evaluation system and an intelligent control system, the training effect is maximized and the risk is minimized (Figure 2).

### 5.1 Personalized plan formulation

The formulation of personalized programs for modern plateau training has developed to the molecular level. The differentiated program based on HIF gene detection analyzes the polymorphism of hypoxia-sensitive genes such as EPAS1 and EGLN1, and divides athletes into three categories: fast adaption type, standard adaption type, and slow adaption type, and formulates differentiated training plans for each type (Yang et al., 2021; Buroker et al., 2012; Graham and McCracken, 2019; Hanaoka et al., 2012). The EPAS1 gene of fast adaption type athletes (accounting for about 15% of the population) has specific single nucleotide polymorphisms (SNPs), and their hypoxic ventilation response is 30%–50% higher than that of the general population (Voisin et al., 2014). They adopt the “3 + 1” advanced mode (3 days of adaptation +1 day of reinforcement), and safely rise 150–200 m per day at an altitude of 2,500 m. The standard adaption type (accounting for about 70%) adopts the conventional “4 + 2” program, and the daily ascent does not exceed 100 m. The slow adaption type (accounting for about 15%) has abnormal expression of the EGLN1 gene and requires a conservative “5 + 2” program, with the daily ascent controlled within 50 m (Buroker et al., 2012). This classification method based on genetic testing can improve the matching of training programs and contributes to more personalized and effective altitude training strategies.

The intensity grading method guided by the blood lactate-power curve uses a standardized incremental load test (power bicycle or treadmill) to measure the power value corresponding to the 4 mmol/L lactate threshold in an environment simulating an altitude of 2,500 m (Huang et al., 2025; Casado et al., 2023). The test plan is: the initial load is 50W, and it increases by 35W every 2 min, while simultaneously monitoring blood lactate, heart rate and ventilation. According to the test results, the training intensity is scientifically divided into 5 levels (Magalhães et al., 2024; Esteve-Lanao et al., 2007): recovery intensity (<85% lactate threshold power, heart rate controlled at 120–140 bpm, blood lactate <2 mmol/L); basic endurance (85%–100%, heart rate 140–160 bpm, blood lactate 2–4 mmol/L); threshold intensity (100%–110%, heart rate 160–175 bpm, blood lactate 4–6 mmol/L); maximum oxygen uptake intensity (110%–120%, heart rate 175–190 bpm, blood lactate 6–8 mmol/L); super-threshold intensity (>120%, heart rate>190 bpm, blood lactate>8 mmol/L). This grading method can make the control error of training intensity less than 5% (Krishnan et al., 2021).

The circadian rhythm adaptation adjustment plan recommends the “morning training and evening rest” mode based on the changes in the rhythm of melatonin secretion in the plateau environment (usually the phase is shifted forward by 1–2 h) (Almendros-Ruiz et al., 2025; Zisapel, 2018; Augsburger et al., 2025): the main training session is carried out from 8 to 10 in the morning (when the cortisol level is high, the body temperature is in the rising period, and the athletic ability is the best); technical training is arranged from 3 to 5 in the afternoon (when the core temperature reaches the peak and the neuromuscular coordination is the best); avoid strenuous activities after 7 p.m. (melatonin begins to be secreted and the body enters the recovery stage). Studies have shown that this arrangement can enable athletes’ circadian rhythms to complete plateau adaptation within 7–10 days, improve sleep quality by more than 30%, and reduce resting heart rate in the morning by 5–8 bpm (Edwards et al., 2002; Ye et al., 2024).

Individualized plans also need to take into account the athlete’s specific characteristics. Endurance events (such as long-distance running and cycling) focus on improving aerobic capacity, with plateau training accounting for 60%–70%, focusing on developing capillary density (increase by 15%–20%) and mitochondrial function (oxidase activity increased by 30%–40%) (Mølmen et al., 2025). Strength and speed events (such as sprinting and weightlifting) focus on neuromuscular adaptation, with plateau training accounting for 40%–50%, mainly improving fast muscle fiber recruitment (EMG amplitude increased by 10%–15%) and force rate (RFD increased by 8%–12%) (Tomazin et al., 2021; Rong et al., 2025). Each training cycle (usually 4 weeks) must also be dynamically adjusted based on the results of periodic evaluations to ensure that the training load is always in the optimal stimulation range.

### 5.2 Dynamic monitoring and regulation

A real-time training load analysis system employs a modified TRIMP (Training Impulse) model, which integrates multiple plateau-specific parameters based on the traditional heart rate method (Lili et al., 2024). The system utilizes smart wearable devices (sampling frequency ≥50 Hz) to collect real-time data on heart rate variability, blood oxygen saturation, and 3D acceleration, enabling the calculation of training impulse per minute. In high-altitude environments, the algorithm has been significantly optimized. A weighting factor for SpO_2_ has been added: when SpO_2_ is 85%–90%, the load coefficient is multiplied by 1.1; 80%–85% by 1.2; and below 80% by 1.5 (Boos et al., 2022). An altitude correction factor is also introduced, increasing the load coefficient by 0.05 for every 500 m of elevation gain. Additionally, circadian rhythm effects are considered, with training loads multiplied by 1.1 during 3–5 p.m. and reduced to 0.8 after 7 p.m. (Castiglioni et al., 2022). This system enhances the accuracy of training load assessment and helps to reduce the risk of overtraining, thereby improving safety and training effectiveness in plateau environments.

The environmental parameter compensation algorithm integrates real-time monitoring data from multiple sources: temperature (updated every 5 min, accuracy ±0.5 °C), relative humidity (±3%), air pressure (±1 hPa) and UV index. The algorithm establishes a complete environment-load correction model. When the ambient temperature is below 10 °C, the intensity target is automatically reduced by 10% (to avoid cold stress superposition) (Foster et al., 2022). The training volume is reduced by 5% for every 500 m increase in altitude (considering the decrease in oxygen partial pressure). When the relative humidity exceeds 70%, the rest time between groups is extended by 20% (to promote heat dissipation) (Périard et al., 2021). When the UV index is >8, the outdoor training time is shortened by 30% (to prevent skin damage). This system can help reduce the risks of overtraining caused by environmental factors and improve the quality of training.

The machine learning-based effect prediction platform uses the long short-term memory (LSTM) neural network architecture to establish a personalized prediction model by analyzing three categories of historical data (Zhang et al., 2022): 1) training data (including plain and plateau training records, load characteristics, and recovery status); 2) physiological data (morning heart rate, blood oxygen, HRV, blood indicators); 3) performance data (special test scores, technical indicators) (Sun et al., 2024). The model automatically updates the weight every 24 h, can predict the training effect 3–5 days in advance (with an accuracy of 75%–85%), and gives optimization suggestions, such as “adding 1 day of recovery period” or “adjusting the altitude by 300 m”. The platform also has an anomaly detection function. When the prediction error exceeds 15%, the cause analysis module is automatically triggered to check for possible interference factors (Bolten, 2024). The similar LSTM-based prediction models and implementations have been reported in sports performance and physiological data analytics (Yang, 2025; Pham, 2021; Sun et al., 2023). Readers can also explore open-source frameworks and examples for time-series prediction, such as TensorFlow (https://www.tensorflow.org/), PyTorch (https://pytorch.org/), and Keras example repositories (https://github.com/keras-team/keras/tree/master/examples), which provide practical guidance for implementing LSTM-based effect prediction platforms.

The dynamic control system also introduces multimodal biofeedback technology. Wireless surface electromyography (sEMG) is used to monitor the activation level and fatigue state of the target muscles (a decrease in median frequency of >15% indicates fatigue) (Taelman et al., 2011). Near-infrared spectroscopy (NIRS) is used to observe local muscle oxygenation in real time (it is recommended to adjust the intensity when the tissue oxygenation index is <40%) (Bovolon et al., 2025). Electroencephalography (EEG) is combined to assess the degree of central fatigue (an increase in theta wave power of more than 20% indicates the need for rest) (Sul et al., 2025). These biological signals are integrated and analyzed with training load data to achieve truly ‘intelligent’ training control, which enhances the protection of the neuromuscular system.

### 5.3 Innovation in emergency response

The intelligent descent decision system uses a multi-objective optimization algorithm and integrates six types of real-time data: 1) GIS geographic information (terrain elevation, path distance, slope); 2) meteorological data (temperature, wind speed, precipitation probability); 3) medical monitoring indicators (SpO_2_, heart rate, blood pressure, state of consciousness); 4) resource distribution (location of medical points, availability of transportation); 5) patient characteristics (age, weight, history of high altitude disease); 6) team conditions (number of escort personnel, equipment status) (Velasquez and Alvarez-Alvarado, 2021). The system calculates the optimal descent path based on the Dijkstra algorithm, considering factors including: altitude descent rate (controlled at 300–500 m/h), transfer time (target <2 h), and risk factor (avoiding dangerous terrain such as cliffs) (Parajuli et al., 2023). When the patient’s SpO_2_<70% or HACE symptoms occur, the system can generate 3 sets of alternative plans within 30 s (such as “descending 800 m from the east road to the medical station” or “rapidly descending 500 m on the west trail”) and give a recommended plan (comprehensive score>90 points) (Garg et al., 2021). During the descent, the system monitors the patient’s condition in real time through GPS and wearable devices, automatically assesses the patient every 100 m of descent, and dynamically adjusts the route and strategy when necessary.

The new portable hyperbaric oxygen chamber uses aviation-grade composite materials (cabin weight <15 kg, load-bearing capacity >150 kg) and intelligent control systems (Luks et al., 2024). Key technological breakthroughs include: 1) rapid deployment (inflation completed within 5 min, reaching 0.3ATA treatment pressure); 2) precise oxygen control (dynamic adjustment of oxygen concentration 90%–95%, fluctuation <2%); 3) comfortable environment (temperature control 20 °C–25 °C, humidity 40%–60%); 4) safety monitoring (real-time display of cabin pressure, oxygen concentration and patient vital signs); 5) remote interconnection (expert consultation through satellite communication) (Kot et al., 2023). Clinical data show that the use of this device to treat acute mountain sickness shortens symptom relief time by more than 50% (HAPE patients are reduced from an average of 4 h–2 h) (Küpper et al., 2022).

Based on a large amount of clinical research data, the drug-training synergistic intervention program has established a refined coordination strategy: 1) During the use of acetazolamide (usually 250 mg bid), appropriately reduce the intensity target by 10%–15%, increase the proportion of recovery training (from 20% to 30%–35%), and pay special attention to supplementing potassium and magnesium electrolytes (80–100 mmol potassium and 400–600 mg magnesium per day) (Bradbury et al., 2020a); 2) During the iron supplementation phase (325 mg ferrous sulfate tid), combined with moderate-intensity continuous training (60%–75% VO_2_max), promote iron absorption and utilization (absorption rate increased by 30%), but avoid high-intensity interval training (reduce free radical production) (McKay et al., 2024; Okazaki et al., 1985); 3) During dexamethasone treatment (4 mg q6h), focus on maintaining technical training (neuromuscular control), suspend maximum strength training (avoid the risk of tendon injury), and increase protein intake (1.6–2.0 g/kg/d) (Montgomery et al., 1989). This synergistic program can reduce the incidence of drug side effects by 50% (such as acetazolamide-related paresthesia reduced from 25% to 12%), while maintaining more than 80% of the expected training effect.

The emergency response system has established a complete drill system, and conducts full-element simulation drills twice a quarter (Eales and Kruger, 2024; Li J. et al., 2024). The drill content includes: 1) scenario simulation (setting different altitudes, weather conditions and types of injuries); 2) role division (clarifying job responsibilities such as medical treatment, escort, and liaison); 3) equipment inspection (testing the reliability of all first aid equipment); 4) process assessment (timing the entire process from the discovery of symptoms to the completion of treatment). A review meeting is held after each drill to analyze the response time (target <60 min), operational standardization, and team cooperation, and continuously improve the emergency plan. The system also established a green channel with tertiary hospitals in low-altitude areas to ensure that critically ill patients can receive advanced life support within 6 h, significantly improving the safety level of plateau training (Luks et al., 2024).

## 6 Discussion

As an important means to improve sports performance, altitude training has achieved remarkable results but also faces many controversies and challenges. With the rapid development of science and technology and the continuous progress of sports medicine, the field of altitude training is undergoing profound changes, which also points the way for future research.

There are two most controversial issues in the field of plateau training. These disputes not only involve the scientific nature of the training effect, but also the boundaries of sports ethics. The ethical issues of drug intervention have attracted much attention in recent years, especially the use of drugs such as EPO. Although EPO can effectively increase hemoglobin concentration and improve oxygen transport capacity, its abuse may lead to a sharp increase in blood viscosity (HCT>55%), increasing the risk of serious cardiovascular events such as thrombosis and myocardial infarction (Thevis et al., 2024). Several clinical studies and meta-analyses have quantified the effects of EPO and high-altitude exposure on hemoglobin concentration and erythropoietic response, highlighting significant individual variability and the potential risks of excessive EPO use, such as increased hematocrit and cardiovascular complications, emphasizing the need for controlled usage and monitoring (Płoszczyca et al., 2018; Dragos et al., 2022; Saugy et al., 2022). What is more worrying is that the emergence of some new hemoglobin regulators (such as HIF stabilizers) has made drug intervention more covert, posing a huge challenge to anti-doping work (Natale et al., 2022; Janssens et al., 2024). The World Anti-Doping Agency (WADA) has to continuously update the banned list, but detection technology often lags behind the development of new drugs (De Wilde et al., 2021; Oleksak et al., 2024). This not only undermines the fairness of sports competitions, but also poses a serious threat to the health of athletes.

Another ongoing focus of debate is the comparison of the effects of altitude training and simulated hypoxia training. Supporters of traditional altitude training emphasize that the real altitude environment (such as 1800–2,500 m above sea level) can induce more comprehensive physiological adaptations, including adaptive changes in pulmonary artery pressure and increased muscle capillary density, which are difficult to fully replicate in a simulated hypoxic environment. Research data show that after 4 weeks of real altitude training, the increase in athletes’ VO2max (4%–8%) is significantly higher than that of simulated hypoxia training (2%–4%) (Saugy et al., 2014; Fentaw et al., 2025). Although simulated hypoxia can achieve some aerobic adaptations, it often fails to induce comparable vascular and hematological changes, highlighting the limitations of current simulation technologies. However, advocates of simulated hypoxia training point out that modern hypoxia tents and training cabins can accurately control oxygen concentration (simulating altitudes up to 4,500 m) and avoid common negative factors in altitude environments (such as ultraviolet radiation, extreme weather, etc.) (Saugy et al., 2014; Humberstone-Gough et al., 2013). More importantly, simulated training can implement flexible programs such as “live high and train low”, which can improve the quality of training by 15%–20% (Huang et al., 2023; Westmac et al., 2022).

The core of this debate lies in the trade-off between the “specificity” and “controllability” of training effects. The contrasting evidence from real vs. simulated hypoxia studies highlights the continued use of both approaches in practice and supports the rationale for developing hybrid strategies to balance physiological adaptations with training safety and convenience (Behrend et al., 2022; Lizamore and Hamlin, 2017; Albertus-Cámara et al., 2022). There is no clear conclusion yet, but more and more training institutions are beginning to adopt a hybrid model that combines the advantages of both. Conceptually, these hybrid strategies could be applied within a microcycle by varying hypoxic exposure during sleep, exercise, or recovery periods. For instance, athletes may avoid ‘live high’ during certain phases to preserve sleep quality, deliberately use post-high-intensity training hypoxia twice per week to enhance anaerobic adaptations, or perform low-intensity hypoxic running (<60% VO_2_max) on specific days to minimize negative impacts on training quality. Although such applications are not yet standardized, they illustrate the potential for periodized hypoxia strategies tailored to different training goals.

The future development of plateau training may focus on two frontiers: breakthroughs in personalized prediction technology and innovations in intelligent monitoring systems. Important progress is being made in the study of gene markers to predict susceptibility to mountain sickness. Genome-wide association studies (GWAS) have identified multiple gene loci associated with plateau adaptation, such as the rs13419896 locus of the EPAS1 gene and the rs1769792 locus of the EGLN1 gene (Li X. et al., 2024; Chen et al., 2014). These gene markers can accurately predict the type of individual response to plateau training (rapid adaptation, standard adaptation, or slow adaptation), with a prediction accuracy of 75%–85%. In the next 5 years, a prediction model based on a polygenic risk score is expected to be put into clinical application, allowing athletes to understand their optimal adaptation altitude and expected adaptation time before training (Wang et al., 2024; Li X. et al., 2024). This personalized prediction can not only improve training results, but also significantly reduce the risk of mountain sickness (estimated to be reduced by 30%–40%).

Real-time monitoring technology for smart wearable devices is undergoing a revolutionary development. The next-generation of monitoring devices will integrate more physiological parameters, including non-invasive hemoglobin monitoring (error <5 g/L), brain oxygen saturation monitoring (prefrontal region), muscle oxygenation status (NIRS technology), etc. These devices can achieve real-time data transmission through 5G/6G networks, and combined with artificial intelligence algorithms, can predict the occurrence of acute mountain sickness 6–12 h in advance (accuracy >90%) (Ma et al., 2023). Of particular note is the development of wearable microfluidic chips, which can monitor key indicators such as lactate and electrolytes in real time through sweat analysis, providing immediate basis for training adjustments (Lin et al., 2020; Sankhala et al., 2021). It is expected that by 2028, these intelligent systems will bring the scientificity and safety of plateau training to a new level, and individual differences in training effects are expected to be reduced by more than 50%. In addition, the application of virtual reality (VR) technology will also change the way of plateau training (Chander et al., 2021). Athletes can use VR devices to simulate the plateau environment in low-altitude areas for psychological adaptation training. This “psychological pre-adaptation” has been shown to reduce 30% of the symptoms of altitude sickness.

## 7 Conclusion

Scientific practice of plateau training shows that scientific adaptation and individualized programs are the core principles for preventing mountain sickness and improving training effects. The use of a scientific “climb high and sleep low” strategy to control the daily vertical ascent to less than 300 m can significantly reduce the incidence of acute mountain sickness from 60% to less than 15%. This adaptive training not only improves the blood oxygen saturation index from the initial 80%–85% to 88%–92%, but more importantly, it promotes the rational secretion of erythropoietin and the adaptive proliferation of capillaries. The individualized program based on genetic testing achieves precise customization of training programs by analysing the polymorphic characteristics of hypoxia-sensitive genes such as EPAS1 and EGLN1, reducing the individual differences in training effects by 40% and reducing the risk of overtraining by 35%. Especially for people with slow adaptation, the conservative “5 + 2” program can increase their training success rate from 50% to 85%.

The application of modern intelligent monitoring technology provides an important guarantee for the safety of plateau training. The early warning system integrating multi-parameter real-time monitoring and artificial intelligence algorithms can predict more than 85% of the risk of acute mountain sickness 12–24 h in advance, reducing the incidence of high-altitude pulmonary edema and cerebral edema by 60% and 75%, respectively. In the future, it is necessary to establish an interdisciplinary plateau training collaboration system, integrate professional knowledge in multiple fields such as physiology, sports training, clinical medicine, bioengineering and data analysis, and jointly formulate standardized safety specifications. The research focus should shift to the exploration of the molecular mechanism of gene-environment interaction, as well as the application and development of innovative technologies such as wearable devices and virtual reality, to provide more scientific and effective guidance for plateau activities for all kinds of people.
